# Middle school students’ mental unwellness and academic performance in China: The effects of parental involvement

**DOI:** 10.1371/journal.pone.0294172

**Published:** 2023-11-09

**Authors:** Keqiao Liu

**Affiliations:** School of Public Finance and Public Administration, Jiangxi University of Finance and Economics, Nanchang, Jiangxi, China; University of the Witwatersrand, SOUTH AFRICA

## Abstract

This study investigated the relationship between the mental unwellness of middle school students in China and their academic performance in the subjects of Chinese, mathematics, and English. Additionally, this study explored the potential ameliorating effects of parental involvement variables (parental non-academic activity involvement and parent-child communication) on the adverse impact of mental unwellness on academic performance. The examination of the effects of parental involvement also considered the differential effects of involvement by mothers and fathers. This study utilized national longitudinal representative data from the China Education Panel Survey (CEPS). Findings of the two-level Hierarchical Linear Modeling (HLM) analyses indicated that mental unwellness in Grade 7 negatively impacted academic performance in the aforementioned subjects in Grade 8. Nonetheless, these adverse impacts were alleviated when the parental involvement variables were taken into account. Furthermore, the results revealed that mother-child communication and father-child communication had moderating effects on the negative relationship between mental unwellness and academic performance in Chinese and English, respectively. This study contributes to the existing literature by shedding light on the beneficial effects of parental involvement and highlighting the differential involvement of mothers and fathers.

## Introduction

Numerous studies have established a correlation between mental unwellness and academic difficulties in children and adolescents [1–4]. On the other hand, as parents play a crucial role in the support of middle school students [5], the literature indicates that parental involvement is positively linked to both mental health [6–8] and academic success [9, 10] of children and adolescents. Hence, it can be postulated that parental involvement may alleviate the deleterious impact of mental unwellness on academic performance. Nevertheless, this effect of parental involvement is perplexed by its multidimensional nature, as different types of parental involvement exhibit varying relationships with the psychological and academic well-being of children and adolescents [9–12]. This complexity is further compounded by whether the types of parental involvement are perceived by children or their parents [11, 12] and whether the types of parental involvement are provided by mothers or fathers [13–15]. Given prior research suggests a stronger effect of children’s perception than parents’ perception [12, 16], this study investigated the potential beneficial effects of different types of parental involvement perceived by children on the academic performance of middle school students. Additionally, this study considered the effects of mother involvement and father involvement utilizing available data.

The investigation into the impact of social support has led to the formulation of two models—the main-effect model and the buffering model (also known as the buffering hypothesis) [17, 18]. The main-effect model posits that social support can lead to people’s enhanced well-being, independent of adverse circumstances. In comparison, the buffering model assumes that social support benefits people only (or primarily) when they are exposed to stressors. Given the similarities between parental involvement and parental support [19–21] and parental support being a component of social support, this study examined the effects of different types of parental involvement on the academic performance of middle school students utilizing the aforementioned models. Specifically, to test the main-effect model, this study analyzed the direct relationships between types of parental involvement and student academic performance. It was hypothesized that positive relationships would be detected and the inverse effect of mental unwellness would be reduced. Then, to test the buffering model, this study examined the interaction effects of different types of parental involvement and mental unwellness on the academic performance of Chinese middle school students. It was expected that a reduction in the negative relationship between mental unwellness and academic performance would be observed among students who received higher levels of parental involvement.

This study conceptualizes parental involvement as the active participation of parents in the educational and non-educational development of their children [11, 22, 23]. Based on this definition, the data employed in this study enabled an examination of two types of parental involvement, namely, parental non-academic activity involvement and parent-child communication. Specifically, parental non-academic activity involvement refers to parents participating in their children’s extracurricular activities, which aligns with the “support nonacademic development” category of parental involvement proposed by Kim et al. [24]. On the other hand, parent-child communication refers to children sharing factual and emotional information with their parents [25]. While the data do not allow for testing the effects of parental non-academic activity involvement by mothers and fathers separately, it offers the chance to examine the effects of mother-child communication and father-child communication as components of parent-child communication. In the meantime, the mental unwellness of middle school students was evaluated using measures provided by the China Education Panel Survey (CEPS). The academic performance of the middle school students was assessed through their test scores in the subjects of Chinese, Mathematics, and English. In general, this study contributes to the understanding of the beneficial effects of parental involvement in addressing the detrimental repercussions of mental unwellness. Moreover, beyond the recognition of parental involvement as a multidimensional construct, this study augments our understanding of the differential effects of mother involvement and father involvement. The research questions addressed in this study are as follows.

1. What are the relationships between the mental unwellness of Chinese middle school students and their academic performance in the subjects of Chinese, mathematics, and English?
2. Can the adverse effects of mental unwellness be alleviated or eliminated by types of parental involvement?
3. Can different types of parental involvement moderate the relationships between middle school students’ mental unwellness and academic performance in Chinese, mathematics, and English?

## Literature review

### Students’ mental health and their academic performance

Researchers have oftentimes demonstrated a positive relationship between the mental unwellness of children and adolescents and their academic difficulties, though the direction of this relationship remains equivocal [1–4, 26–28]. Some studies indicate that mental unwellness predicts academic difficulties. For example, Deighton et al. [3] state that higher levels of internalizing symptoms can lead to subsequent reductions in academic attainment among secondary students. Similarly, Agnafors et al. [1] indicate that mental health problems at preschool age can result in academic difficulties in English and mathematics at age 12, while mental health problems at age 12 increase students’ later risk of non-eligibility to higher education. A possible cause of mental unwellness in children and adolescents can be their exposure to childhood trauma (e.g., abuse and neglect), which then affects their academic performance [4, 29]. Researchers who support the impact of mental health problems on academic performance, rather than vice versa, refer to this phenomenon as adjustment erosion [3, 30, 31] or social selection [1, 32].

Some other researchers have indicated that the academic difficulties experienced by children and adolescents serve as predictors of their mental unwellness. For instance, Moilanen et al. [30] state that poor academic competence, such as difficulty in completing class assignments within a given time frame, at ages 8 and 10 is predictive of mental unwellness at ages 10 and 11. Similarly, the results of Verboom et al. [33] indicate that poor academic performance among Dutch adolescent girls can lead to depressive problems. More recently, Metsapelto et al. [34] assert that better mathematics performance in Grade 5 predicts higher self-esteem in Grade 6, which in turn predicts fewer internalizing and emotional problems in Grade 7. This phenomenon has been referred to as academic incompetence [3, 30, 31] or social causation [1, 32]. It suggests that the academic difficulties faced by children and adolescents may produce or exacerbate their mental unwellness, such as stress and depression.

To address the issue of reverse causality, Fletcher [35] examines the causal relationship between adolescent depression and educational attainment. The results indicate that depressive symptoms negatively impact US adolescents’ educational attainment. Among recent studies that aim to unravel the connection between academic performance and mental health, the hypothesis of adjustment erosion/social selection has received more support than the hypothesis of academic incompetence/social causation [1, 3, 31]. Specifically, Agnafors et al. [1] find that mental health problems among Swedish children and adolescents can result in subsequent academic difficulties, but not the reverse. Likewise, Deighton et al. [3], based on an examination of UK secondary students, reveal that internalizing symptoms predict later academic attainment, but prior academic attainment is not associated with later internalizing symptoms. Additionally, Zhang et al. [31] state that higher levels of depressive symptoms among Chinese students in Grade 5 and Grade 6 can predict lower academic performance in Grade 6 and Grade 7, respectively. In comparison, the relationship between prior academic performance and later depressive symptoms is only specific between Grade 6 and Grade 7. Based on the aforementioned, this study investigated whether the mental unwellness of Chinese middle schoolers can predict their academic performance in Chinese, mathematics, and English. More importantly, this study explored the role of different types of parental involvement in alleviating and moderating the effects of mental unwellness on academic performance.

### Parental involvement and its relationships with the mental health and academic performance of children and adolescents

Higher levels of parental involvement are generally associated with lower levels of mental unwellness in children and adolescents [6–8, 16]. For instance, Pengpid and Peltzer [7] reveal that parental involvement is inversely correlated with anxiety among adolescents from seven Pacific Island countries. On the other hand, Thomas et al. [16] indicate that higher levels of parental involvement are linked to Belgian middle school students’ higher levels of school well-being (e.g., how happy students are at school). Nevertheless, it is worth noting that different types of parental involvement may show varied relationships with the mental unwellness of children and adolescents. For example, the findings of Liu et al. [11] demonstrate that higher levels of children’s perception of parent-child communication are associated with middle schoolers’ lower levels of depression during the staying-at-home stage of the COVID-19 pandemic, while higher levels of children’s perception of parental academic involvement are correlated with middle schoolers’ higher levels of depression. Although the study of Liu et al. [11] should be considered in the context of the staying-at-home stage of the COVID-19 pandemic, it suggests the necessity of examining parental involvement as a multidimensional construct to gain a more comprehensive understanding of its beneficial effects. The two types of parental involvement being examined in this study are parental non-academic activity involvement and parent-child communication. From the perspective of ecological systems theory [36], these are the types of parental involvement that children have direct rather than indirect (e.g., parent-teacher communication) experiences.

Parental involvement has long been believed by researchers to be positively associated with their children’s academic performance [9, 10, 16, 23]. Based on their meta-analysis, Fan and Chen [10] state that higher levels of parental involvement are related to better academic performance in students. Similarly, Boonk et al. [9] assert that parental involvement is positively associated with the academic performance of children and adolescents. Besides, Thomas et al. [16] point out in their research that children’s perception of parental involvement, rather than parents’ perception, is positively related to students’ academic performance. This justifies this study’s approach of examining children’s perception of parental involvement.

On the other hand, it is important to acknowledge the multifaceted nature of parental involvement when researchers explore its impact on the academic performance of children and adolescents [9, 10, 37]. According to Liu et al. [37], children’s perception of parent-child communication and parental daily involvement are positively associated with student behavioral, emotional, and cognitive engagement, while children’s perception of parental academic involvement has weak associations with these dimensions of student engagement. Given that student engagement is positively associated with academic performance [38, 39], the aforementioned findings suggest that when researchers examine the beneficial effects of parental involvement on student academic performance, they should consider different types of parental involvement.

Prior studies suggest that disparities exist between father involvement and mother involvement [13–15]. For example, scrutinizing adolescents in Taiwan, Hsu et al. [13] indicate that mother involvement is positively associated with academic performance, while father involvement is not related to academic performance. In comparison, based on their meta-analysis, Kim and Hill [14] state that the link between father involvement and academic performance is comparable to the link between mother involvement and academic performance, although father involvement tends to be lower. The findings from these studies highlight the lack of consensus regarding the effects of mother involvement and father involvement on the academic performance of children and adolescents. Combining this with the multidimensional nature of parental involvement, this study attended to parent-child communication by distinguishing it as mother-child communication and father-child communication. Unfortunately, due to data unavailability, an examination of parental non-academic activity involvement by mothers and fathers was not possible.

## Methods

### Data

This study utilized secondary data from the China Education Panel Survey (CEPS), which was approved by the Renmin University of China. It is a nationally representative longitudinal dataset collected by the National Survey Research Center at Renmin University of China. The CEPS currently comprises two waves of publicly available data, which do not contain any information that can identify individual participants. Specifically, Grade 7 (N = 10,279) and Grade 9 (N = 9,208) students from 112 Chinese schools were first surveyed in the academic year 2013–2014 (Wave 1), and then those Grade 7 (N = 9,449) students were followed up in the academic year 2014–2015 (Wave 2). This study focused on the students who were in Grade 7 in Wave 1, as only they were followed up in Wave 2.

This study implemented listwise deletion and weighting (*w1w2sweight*). Specifically, the weight being utilized was a panel weight calculated by the CEPS to adjust for student attrition between wave 1 and wave 2. Notably, the CEPS did not provide any school-level weights. After weighting, the results indicate that 49% of the students were female, 87.1% were Han (the predominant ethnic group in China), and 37.4% were from the center of the city/town (see Table 1 for more details). Similarly, data from the Ministry of Education of the People’s Republic of China [40] show that in 2013, 46.5% of the Grade 7 students were female (N_female_ = 6,962,278; N_total_ = 14,972,216), 88.9% were Han (N_Han_ = 13,309,009), and 32.2% were from urban areas (N_urban_ = 4,817,167). Further, recent data provided by the Ministry of Education of the People’s Republic of China [41] show that, in 2020, 46.4% of the Grade 7 students were female (N_female_ = 7,569,306; N_total_ = 16,324,100), 88.1% were Han (N_Han_ = 14,378,010), and 39.5% were from urban areas (N_urban_ = 6,453,318). In general, the data used in this study exhibit similarities with the demographic characteristics of the population in 2013 and 2020.

**Table 1:** Variable description.

Variable Name	Variable Description	Mean	Min	Max	SD	Cronbach’s α
**Level-1 variables**						
Chinese	Chinese test scores in Wave 2	68.436	7.67	98.33	15.730	
Math	Math test scores in Wave 2	63.025	0.00	136.67	26.985	
English	English test scores in Wave 2	59.112	0.00	106.00	24.957	
Mental unwellness	Based on 5 items, i.e., 1) feeling blue in the last seven days; 2) depressed in the last seven days; 3) unhappy in the last seven days; 4) not enjoying life in the last seven days; 5) sad in the last seven days. (Wave 1)	2.011	1.00	5.00	0.741	0.827
Parental non-academic activity involvement	Based on 3 items, i.e., 1) playing sports with parents; 2) visiting museums, zoos, science museums, etc. with parents; 3) going out to watch movies, shows, sports games, etc. with parents. (Wave 1)	2.501	1.00	6.00	1.346	0.744
Mother-child communication	Based on 5 items, i.e., 1) discussing with mom about things that happened at school; 2) discussing with mom about the relationship with friends; 3) discussing with mom about the relationship with teachers; 4) discussing with mom about one’s feelings; 5) discussing with mom about worries and troubles. (Wave 1)	2.137	1.00	3.00	0.549	0.790
Father-child communication	Based on 5 items, e.g., 1) discussing with dad about things that happened at school; 2) discussing with dad about the relationship with friends; 3) discussing with dad about the relationship with teachers; 4) discussing with dad about one’s feelings; 5) discussing with dad about worries and troubles. (Wave 1)	1.923	1.00	3.00	0.561	0.807
Chinese	Standardized Chinese test scores in Wave 1	71.175	9.96	97.60	8.971	
Math	Standardized math test scores in Wave 1	71.062	18.68	145.11	9.570	
English	Standardized English test scores in Wave 1	71.172	11.35	93.42	9.343	
Female (vs. male)	Students’ gender (Wave 1)	0.490	0	1		
Han (vs. minorities)	Being an ethnic majority or not (Wave 1)	0.871	0	1		
Only Child (vs. ≥ one)	Being the only child or not (Wave 1)	0.345	0	1		
Parent postsecondary education (vs. no)	At least one parent holding a postsecondary education degree (Wave 1)	0.131	0	1		
**Level-2 variables**						
Outskirts of the city/town	School location (vs. center of the city/town) (Wave 1)	0.107	0	1		
Rural-urban fringe zone		0.152	0	1		
Towns outside of the city/town		0.179	0	1		
Rural areas		0.188	0	1		
Public school	School type (vs. private) (Wave 1)	0.929	0	1		

Notes: 1. The leve1-1 sample size = 7,924 and the level-2 sample size = 112; 2. Since it is possible to earn extra points, the maximum scores in Chinese, mathematics, and English can be more than 100; 3. Values in the table were weighted by *w1w2sweight*.

### Variables

#### Academic performance in Chinese, mathematics, and English in Grade 8 (Wave 2)

The academic performance of middle school students in the subjects of Chinese, mathematics, and English in Grade 8 came from their mid-term raw scores in Chinese (*w2chn*), mathematics (*w2mat*), and English (*w2eng*) of the 2014 fall semester. In China, the academic grading system often employs a scale of 0 to 100, where the pass grade is usually 60. Thus, to account for variations in the maximum scores of the academic subjects across different regions and schools, this study converted the full marks of mid-term tests to 100, using the raw full marks offered by the CEPS (Chinese: *w2upchn*; math: *w2upmat*; English: *w2upeng*). Specifically, the raw full marks of Chinese (*w2upchn*) included 4 categories, i.e., 100 (40.2% of the 112 schools), 120 (38.4%), 130 (2.7%), and 150 (18.8%). The full marks of mathematics (*w2upmat*) included 4 categories, i.e., 100 (41.1% of the 112 schools), 120 (37.5%), 130 (2.7%), and 150 (18.8%). Then, the full marks of English (*w2upeng*) contained 5 categories 100 (42.9% of the 112 schools), 120 (37.5%), 130 (0.9%), 140 (0.9%), and 150 (17.9%). The weighted mean scores for Chinese, mathematics, and English were 68.436 (SD = 15.730, Skewness = -1.061, Kurtosis = 1.276), 63.025 (SD = 26.985, Skewness = -0.459, Kurtosis = -0.980), and 59.112 (SD = 24.957, Skewness = -0.275, Kurtosis = -1.099), respectively (see Table 1).

#### Mental unwellness

The CEPS used five items (*a1801*, *a1802*, *a1803*, *a1804*, *a1805*) to assess the mental unwellness of Grade 7 students (Wave 1). A sample item is “feeling blue in the last seven days,” with responses on a 5-point scale (1 = never, 2 = seldom, 3 = sometimes, 4 = often, 5 = always). While the CEPS does not provide information on the origin of its mental unwellness scale, its items are similar to certain items of the Center for Epidemiologic Studies Depression Scale (CES-D). For example, a CES-D sample item is “I felt that I could not shake off the blues even with help from my family or friends during the past week.” Studies such as Jiang et al. [42] and Jiang et al. [43] adopted the CEPS mental health scale in their research. The Cronbach’s alpha of mental unwellness in Grade 7 students was 0.827. A composite variable was generated by taking the mean of the five items, with a higher value indicating a greater level of mental unwellness (mean = 2.011, SD = 0.741, Skewness = 0.995, Kurtosis = 1.710) (see Table 1).

#### Parental non-academic activity involvement

Based on Kim et al.’s [24] research, this study perceived parental non-academic activity involvement as the type of parental involvement that supports the non-academic development of children (e.g., supporting children’s interests). Besides, we considered the items of parental daily involvement that Liu et al. [37] utilized, e.g., parents supporting their children to participate in extracurricular activities. In the end, the parental non-academic activity involvement variable was created based on children’s perceptions of three items (e.g., “playing sports with parents”) collected in Grade 7 (*b2804*, *b2805*, *b2806*). Responses were on a 6-point scale (1 = never, 2 = once a year, 3 = once every half year, 4 = once a month, 5 = once a week, 6 = more than once a week). Exploratory Factor Analysis (EFA) extracted one dimension (parental non-academic activity Involvement). The Cronbach’s alpha was 0.744. A derived variable was obtained based on the mean of the three items, with a higher value indicating a higher level of parental non-academic activity involvement (mean = 2.501, SD = 1.346, Skewness = 0.709, Kurtosis = -0.187) (see Table 1).

#### Parent-child communication

Children’s perception of parent-child communication consists of mother-child communication and father-child communication. The CEPS used five items to measure mother-child communication (*b24a1*, *b24a2*, *b24a3*, *b24a4*, *b24a5*) and father-child communication (*b24b1*, *b24b2*, *b24b3*, *b24b4*, *b24b5*) among Grade 7 students, respectively. The items used by CEPS reflected the measurement of factual and emotional information exchange between children and their parents [25]. A sample item of mother-child communication was “discussing with mom about things that happened at school,” with answers on a 3-point scale (1 = never, 2 = sometimes, 3 = often). EFAs extracted one dimension (parent-child communication) for mothers and fathers, respectively. The Cronbach’s alpha for mother-child communication was 0.790, while the Cronbach’s alpha for father-child communication was 0.807. Derived variables were separately created based on the mean of the five items, with a higher value indicating a higher level of mother-child communication (mean = 2.137, SD = 0.549, Skewness = -0.204, Kurtosis = -0.790) and father-child communication (mean = 1.923, SD = 0.561, Skewness = 0.061, Kurtosis = -0.831) (see Table 1).

#### Control variables

The level-1 (student level) control variables included students’ academic performance in Chinese (*stdchn*, mean = 71.175, SD = 8.971, Skewness = -1.032, Kurtosis = 2.087), mathematics (*stdmat*, mean = 71.062, SD = 9.570, Skewness = -0.710, Kurtosis = 0.543), and English (*stdeng*, mean = 71.172, SD = 9.343, Skewness = -1.039, Kurtosis = 1.486) in Grade 7 (Wave 1), students’ gender (*a01*, 49% females), student ethnicity (*a03*, 87.1% Han vs. 12.9% other minorities), whether a student is the only child (*b0*1, 34.5% only child), and parents’ educational level (*b06*, *b07*, 13.1% of the students having at least one parent holding a postsecondary education degree) (see Table 1).

The Level-2 (school level) control variables were school location (*pla23*, 37.4% center of the city/town, 10.7% outskirts of the city/town, 15.2% rural-urban fringe zone, 17.9% towns outside of the city/town, 18.8% rural areas) and school type (*pla01*, 92.9% public schools; 7.1% private schools). Notably, among the 112 schools being surveyed by the CEPS, there were 104 public schools, 1 private school subsidized by the government, 5 ordinary private schools, and 2 private schools for children of migrant workers. Due to the small sample size of the different types of private schools, this study grouped private schools together (see Table 1).

### Data analysis

Using SPSS 26 and HLM 6, this study conducted both descriptive and inferential analyses to address the three research questions. To answer research question 1, this study first ran weighted Pearson correlations between mental unwellness and academic performance in Chinese, mathematics, and English in Grade 8. Then, after considering the nested structure of the data and controlling for other variables, two-level Hierarchical Linear Modeling (HLM) analyses were employed to predict students’ academic performance in Grade 8. To answer research question 2, based on the HLM analyses, this study compared the effects of mental unwellness on academic performance in Chinese, mathematics, and English with and without controlling for parental involvement variables. Finally, to answer research question 3, this study carried out HLM analyses with interaction effects of different types of parental involvement and mental unwellness. The full HLM model is presented below.

### Level 1 (student level)

$$\begin{array}{c} Y=\beta_{0}+\beta_{1}\;*\;Mental+\beta_{2}\;*\;Nonacademic+\beta_{3}\;*\;MCOM+\beta_{4}\;*\;FCOM+\beta_{5}\;*\;\\ Mental\;*\;Nonacademic+\beta_{6}\;*\;Mental\;*\;MCOM\;\beta_{7}\;*\;Mental\;*\;FCOM+\\ \beta_{8}\;*\;ACA1+\beta_{9-12}\;*\;Control+r \end{array}$$

### Level 2 (school level)

$$\beta_{0}=\gamma_{00}+\gamma_{01-04}\;*\;sLocation+\gamma_{05}\;*\;Stype+u_{0}$$

*β*_1_ = γ_10_

…

*β*_12_ = γ_120_

Y represents middle school students’ academic performance in Chinese, mathematics, and English in Grade 8 (Wave2); *Mental* represents the standardized mental unwellness variable; *Nonacademic* represents the standardized parental non-academic activity involvement variable; *MCOM* represents the standardized mother-child communication variable; FCOM represents the standardized father-child communication variable; *Mental* * *Nonacademic* represents the interaction between mental unwellness and parental non-academic activity involvement; *Mental* * *MCOM* represents the interaction between mental unwellness and mother-child communication; *Mental* * *FCOM* represents the interaction between mental unwellness and father-child communication; *ACA1* represents students’ academic performance in Chinese, mathematics, or English in Grade 7 (Wave 1); *Control* represents a set of level-1 control variables (e.g., gender); *sLocation* represents the dummy variables for school location (reference = center of the city/town); *Stype* represents school type (reference = private).

The results of the Intraclass Correlation (ICC) obtained from the fully unconditional model (no predictors) of the HLM indicated the feasibility of using HLMs to address the research questions. The ICC, which is calculated as the ratio of the between-school variance to the total variance, demonstrated that 39.9% of the variance in Chinese test performance among middle school students was attributable to differences between schools ($ICC=\frac{103.959}{103.959+156.478}=0.399$). In terms of mathematics test performance, 23.4% of the variance was explained by differences between schools ($ICC=\frac{171.112}{171.112+559.108}=0.234$). Lastly, 33.9% of the variance in English test performance among students was due to differences between schools ($ICC=\frac{219.891}{219.891+427.899}=0.339$).

## Results

### Correlations among mental unwellness, parental involvement, and academic performance in Grade 8

As shown in Table 2, middle school students’ mental unwellness in Grade 7 was significantly and negatively associated with their academic performance in Chinese (*r* = -0.157, *p* < 0.05), mathematics (*r* = -0.170, *p* < 0.05), and English (*r* = -0.179, *p* < 0.05) in Grade 8, though the correlations were weak. This suggests that higher levels of mental unwellness in Grade 7 were related to lower levels of academic performance in the aforementioned subjects in Grade 8. In comparison, children’s perceptions of parental non-academic activity involvement (Chinese: *r* = 0.151, *p* < 0.05; math: *r* = 0.114, *p* < 0.05; English: *r* = 0.197, *p* < 0.05), mother-child communication (Chinese: *r* = 0.230, *p* < 0.05; math: *r* = 0.194, *p* < 0.05; English: *r* = 0.251, *p* < 0.05), and father-child communication (Chinese: *r* = 0.132, *p* < 0.05; math: *r* = 0.107, *p* < 0.05; English: *r* = 0.127, *p* < 0.05) in Grade 7 were all positively and significantly associated with their academic performance in Chinese, mathematics, and English in Grade 8, although the correlations were weak.

**Table 2:** Correlations among independent and dependent variables.

		1	2	3	4	5	6
1	Chinese (Wave 2)	1					
2	Math (Wave 2)	0.666^***^					
3	English (Wave 2)	0.666^***^	0.712^***^				
4	Mental unwellness	-0.157^***^	-0.170^***^	-0.179^***^			
5	Parental non-academic activity involvement	0.151^***^	0.114^***^	0.197^***^	-0.180^***^		
6	Mother-child communication	0.230^***^	0.194^***^	0.251^***^	-0.171^***^	0.390^***^	
7	Father-child communication	0.132^***^	0.107^***^	0.127^***^	-0.157^***^	0.351^***^	0.614^***^

Notes: 1. Correlations were weighted by *w1w2sweight*; 2. Before weighting the level-1 sample size = 7,924, after weighting the level-1 sample size = 12,638,807

^***^ *p* < 0.001.

In the meantime, the correlations between students’ academic performance in Chinese, mathematics, and English in Grade 8 ranged from 0.666 to 0.712, indicating moderate to strong relationships. Additionally, children’s perception of parental non-academic activity involvement was weakly to moderately associated with children’s perceptions of mother-child communication (*r* = 0.390, *p* < 0.05) and father-child communication (*r* = 0.351, *p* < 0.05). Furthermore, children’s perception of mother-child communication was moderately to strongly related to children’s perception of father-child communication (*r* = 0.614, *p* < 0.05).

### Hierarchical linear models predicting students’ performance in Chinese

After holding constant the control variables, middle schoolers’ higher levels of mental unwellness in Grade 7 were still related to their worse performance in Chinese in Grade 8, β = -0.953, *p* < 0.05 (Table 3, Model 1). After controlling for parental involvement variables, this negative relationship remained, though to a slightly lesser extent, β = -0.891, *p* < 0.05 (Table 3, Model 2). Meanwhile, a comparison of the effects of parental involvement variables revealed that mother-child communication in Grade 7 was positively associated with students’ academic performance in Chinese in Grade 8 (β = 0.657, *p* < 0.05), while parental non-academic activity involvement (β = 0.000, *p* > 0.05) and father-child communication (β = -0.322, *p* > 0.05) were no longer associated with middle schoolers’ performance in Chinese in Grade 8 (Table 3, Model 2).

**Table 3:** Hierarchical linear models predicting students’ academic performance in Chinese, mathematics, and English.

	Chinese		Mathematics		English	
	Model 1	Model 2	Model 1	Model 2	Model 1	Model 2
	Coefficient (*se*)	Coefficient (*se*)	Coefficient (*se*)	Coefficient (*se*)	Coefficient (*se*)	Coefficient (*se*)
**Level-1 Variables**						
Mental unwellness^a^	-0.953 (0.226)^***^	-0.891 (0.213)^***^	-1.411 (0.364)^***^	-1.264 (0.346)^***^	-1.471 (0.362)^***^	-1.316 (0.345)^***^
Parental non-academic activity involvement^a^		0.000 (0.207)		-0.414 (0.316)		-0.044 (0.235)
Mother-child communication^a^		0.657 (0.173)^***^		1.298 (0.285)^***^		1.147 (0.268)^***^
Father-child communication^a^		-0.322 (0.170)		-0.268 (0.324)		-0.207 (0.254)
Interactions						
Mental unwellness^a^ × Parental non-academic activity involvement^a^		0.126 (0.118)		-0.008 (0.256)		-0.131 (0.198)
Mental unwellness^a^ × Mother-child communication^a^		0.296 (0.152)^˄^		0.236 (0.281)		-0.134 (0.296)
Mental unwellness^a^ × Father-child communication^a^		-0.130 (0.132)		0.264 (0.275)		0.456 (0.234)^˄^
Chinese performance in Wave 1	0.860 (0.049)^***^	0.855 (0.050)^***^				
Math performance in Wave 1			1.747 (0.084)^***^	1.739 (0.085)^***^		
English performance in Wave 1					1.616 (0.076)^***^	1.606 (0.076)^***^
Female (vs. male)	3.292 (0.397)^***^	3.181 (0.404)^***^	3.172 (0.629)^***^	2.896 (0.613)^***^	3.834 (0.741)^***^	3.631 (0.719)^***^
Han (vs. minorities)	0.552 (0.704)	0.567 (0.705)	1.095 (1.530)	1.170 (1.557)	0.410 (0.983)	0.433 (1.008)
Only child (vs. more than one child)	0.187 (0.380)	0.194 (0.379)	0.331 (0.589)	0.334 (0.585)	0.109 (0.501)	0.054 (0.493)
Parent postsecondary education (vs. no)	1.400 (0.508) ^**^	1.342 (0.501)^**^	2.947 (0.753)^***^	2.926 (0.741)^***^	2.374 (0.809)^**^	2.221 (0.818)^**^
**Level-2 Variables**						
School Location (vs. center of the city/town)						
Outskirts of the city/town	-2.479 (3.312)	-2.496 (3.293)	-5.193 (6.135)	-5.360 (6.078)	-4.621 (4.817)	-4.747 (4.767)
Rural-urban fringe zone	-6.237 (6.893)	-6.182 (6.832)	-11.392 (5.170)^*^	-11.322 (5.089)^*^	-14.576 (7.068)^*^	-14.471 (6.948)^*^
Towns outside of the city/town	-6.195 (3.922)	-6.134 (3.903)	-6.500 (4.477)	-6.560 (4.417)	-13.920 (4.463)^**^	-13.839 (4.384)^**^
Rural areas	-3.224 (2.512)	-3.131 (2.468)	-7.539 (3.939)	-7.579 (3.888)^˄^	-17.304 (4.842)^**^	-17.122 (4.763)^**^
Public school (vs. private)	-4.194 (3.569)	-4.266 (3.531)	-3.920 (3.173)	-3.798 (3.142)	8.221 (3.654)^*^	8.202 (3.589)^*^
**Random Effect**	Variance Component (*df*)	Variance Component (*df*)	Variance Component (*df*)	Variance Component (*df*)	Variance Component (*df*)	Variance Component (*df*)
*u*_*0*_	100.677 (106)^***^	99.379 (106)^***^	166.730 (106)	163.392 (106)^***^	182.879 (106)^***^	178.605 (106)^***^

Notes: 1. The leve1-1 sample size = 7,924 and the level-2 sample size = 112; 2.

^a^ The variables were standardized; 3. The models were weighted by level-1 panel weight, *w1w2sweight*; 4.

^˄^ ≈ 0.05

^*^ *p* < 0.05

^**^ *p* < 0.01

^***^ *p* < 0.001.

**Fig 1:**
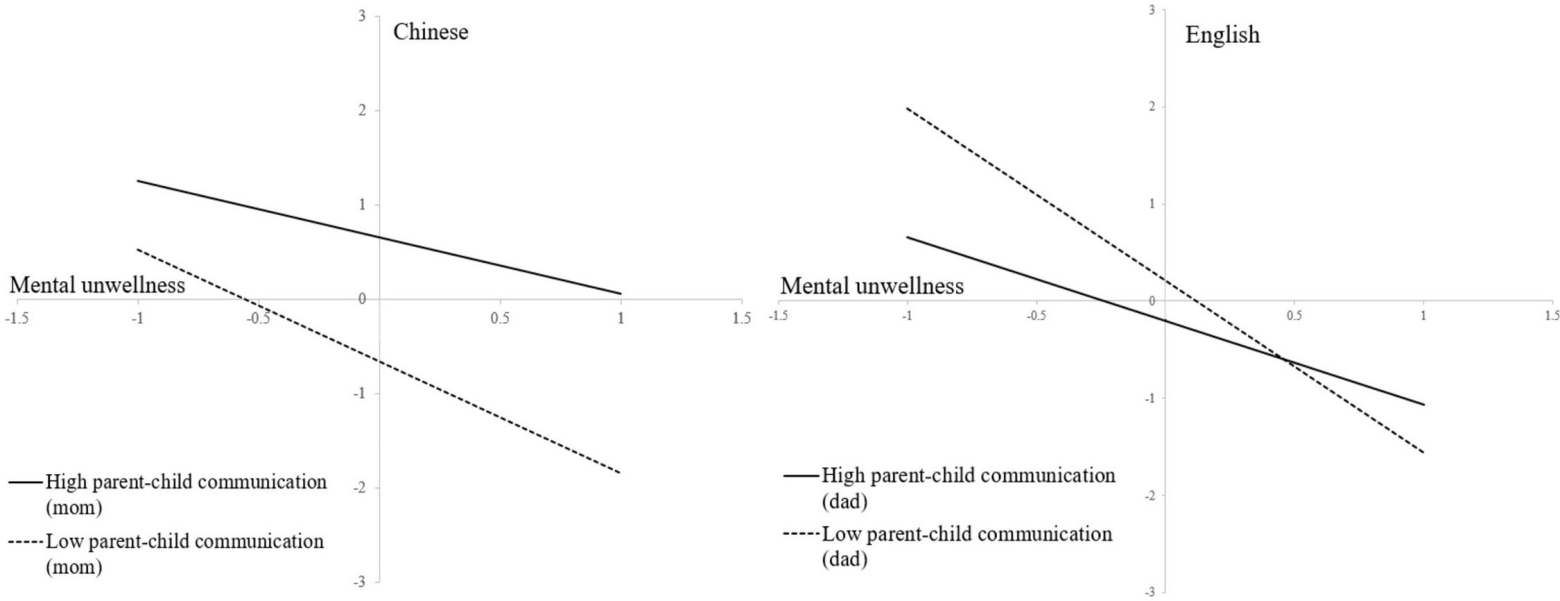
Interactions between parent-child communication and mental unwellness on students’ Chinese and English performance. Notes: 1. The high parent-child communication groups were set at one SD above the mean, while the low parent-child communication groups were set at one SD below the mean; 2. All the other variables and the constants were set at 0.

Notably, the relationship between mental unwellness and academic performance in Chinese varied by the levels of mother-child communication, β = 0.296, *p* < 0.05 (Table 3, Model 2). Specifically, the negative relationship between middle schoolers’ mental unwellness in Grade 7 and their academic performance in Chinese in Grade 8 was weaker for middle schoolers who perceived higher levels of mother-child communication (Fig 1). In comparison, no moderating effects were observed between mental unwellness and either parental non-academic activity involvement (β = 0.126, *p* > 0.05) or father-child communication (β = -0.130, *p* > 0.05).

Based on Model 2, the level-1 predictors explained 46.5% ($\frac{156.478-83.573}{156.478}=0.465$) of the within-school variance in Chinese, while the level-2 predictors accounted for 4.4% ($\frac{103.959-99.379}{103.959}=0.044$) of the between-school variance.

### Hierarchical linear models predicting students’ performance in mathematics

As presented in Table 3 (Model 1), after controlling for variables such as prior performance in mathematics, the inverse relationship between middle schoolers’ mental unwellness in Grade 7 and their academic performance in mathematics in Grade 8 remained (β = -1.411, *p* < 0.05). Further analysis incorporating parental involvement variables revealed that mental unwellness still had a negative association with academic performance in mathematics, β = -1.264, *p* < 0.05 (Model 2). Among the three parental involvement variables considered, only children’s perception of mother-child communication was positively related to academic performance in mathematics (β = 1.298, *p* < 0.05), while neither parental non-academic activity involvement (β = -0.414, *p* > 0.05) nor father-child communication (β = -0.268, *p* > 0.05) were found to be associated with academic performance in mathematics in Grade 8 (Table 3, Model 2).

In the meantime, no interaction effects were detected between parental involvement variables and mental unwellness (Table 3, Model 2). That is, the inverse relationship between mental unwellness and academic performance in mathematics was found to be consistent across varying levels of parental non-academic activity involvement (β = -0.008, *p* > 0.05), mother-child communication (β = 0.236, *p* > 0.05), and father-child communication (β = 0.264, *p* > 0.05). Based on Model 2, the level-1 predictors explained 53.3% ($\frac{559.108-261.312}{559.108}=0.533$) of the within-school variance in mathematics performance, while the level-2 predictors accounted for 4.5% ($\frac{171.112-163.392}{171.112}=0.045$) of the between-school variance.

### Hierarchical linear models predicting students’ performance in English

After controlling for English performance in Grade 7, the results indicated that middle schoolers’ mental unwellness in Grade 7 was still inversely related to their subsequent academic performance in English in Grade 8, β = -1.471, *p* < 0.05 (Table 3, Model 1). Further control for parental involvement variables did not significantly alter this negative relationship, which remained significant, β = -1.316, *p* < 0.05 (Table 3, Model 2). Among the parental involvement variables, when everything else was equal, only children’s perception of mother-child communication was positively associated with English performance in Grade 8, β = 1.147, *p* < 0.05 (Table 3, Model 2).

In regard to the moderation effects, the negative relationship between mental unwellness and English performance was weaker among students who perceived higher levels of father-child communication (β = 0.456, *p* < 0.05). Nonetheless, no moderation effects were found for either parental non-academic activity involvement (β = -0.131, *p* > 0.05) or mother-child communication (β = -0.134, *p* > 0.05) on the aforementioned relationship.

Based on Model 2, the level-1 predictors explained 60.6% ($\frac{427.899-168.441}{427.899}=0.606$) of the within-school variance in English performance in Grade 8, while the level-2 predictors accounted for 18.8% ($\frac{219.891-178.605}{219.891}=0.188$) of the between-school variance. Compared to the models on academic performance in Chinese and mathematics, a greater proportion of the between-school variance in English performance was explained by the school-level variables (i.e., school location and school type).

## Discussion and conclusion

### Chinese middle schoolers’ mental unwellness and their academic performance in Chinese, mathematics, and English

This study, utilizing longitudinal data, demonstrated the existence of adjustment erosion/social selection [1, 3, 30–32]. The results showed that after controlling for factors such as prior academic performance and parental involvement, higher levels of mental unwellness among middle school students in Grade 7 were related to worse academic performance in Chinese, mathematics, and English in Grade 8. As speculated by prior researchers [4, 29], the mental unwellness of middle schoolers may result from their exposure to childhood trauma (e.g., abuse). On the other hand, while this study did not examine the potential impact of academic performance on mental wellness, it is reasonable to assume its presence based on extant studies [30, 33]. Accordingly, the adverse effect of mental unwellness on academic performance in this study may perpetuate or exacerbate the mental unwellness of middle school students, thus trapping students in a vicious circle. Strategies such as providing counseling services to students and their parents may promote students’ mental well-being to address the aforementioned problem. Besides, means such as reforming China’s annually-held high-stakes examinations, i.e., the high school entrance examination (*zhongkao*) and the national college entrance examination (*gaokao*), may help to reduce students’ academic burden, ease students’ academic stress, and alleviate parental educational anxiety, all of which can benefit students’ well-being.

A closer look at the results reveals that the adverse effects of mental unwellness were most pronounced on the academic performance of middle school students in English, followed by mathematics. In comparison, the detrimental impact of mental unwellness on academic performance in Chinese was comparatively less severe. This may result from the fact that Chinese students live in a Chinese immersion environment, where they not only acquire knowledge of Chinese through formal school education but also learn through their everyday lives. In contrast, Chinese students mostly learn English and mathematics through formal education. After entering secondary education, they face an increased difficulty level in academic subjects, which can make them depend more on learning through formal education. Accordingly, the potential adverse impacts of mental unwellness on Chinese students’ academic performance in English and mathematics can be greater than in Chinese. Nevertheless, to gain a more thorough understanding, future researchers should investigate the underlying reasons extensively.

### The effects of parental involvement

The findings of this study indicate that the inclusion of parental involvement variables (parental non-academic activity involvement, mother-child communication, and father-child communication) diminished the negative impact of mental unwellness on middle school students’ academic performance in the subjects of Chinese, mathematics, and English. This implies that parental involvement can alleviate the adverse effects of mental unwellness on student academic performance. However, it should be noted that the parental involvement variables utilized in this study were unable to eliminate the negative impact of mental unwellness. Thus, future research is necessary to examine the effects of other types of parental involvement, as this study was limited by the availability of data and only investigated two types of parental involvement that are not directly academically related [24]. It is plausible that types of parental involvement that are more directly academic-related may exert a greater influence in mitigating the adverse effects of mental unwellness on academic performance. For instance, a study by Yang et al. [12] reveals a positive relationship between parental academic involvement and affective engagement in middle school students. Since affective engagement has been linked to improved academic performance [39], parental academic involvement may benefit students’ academic performance and protect them from the unfavorable impact of mental unwellness. The findings of this study suggest that while educators and other stakeholders emphasize the importance of parental involvement, they should also acknowledge the disparities in the types of parental involvement. Therefore, parental involvement-promoting strategies, such as schools holding workshops for parents, should focus on how to encourage different types of parental involvement.

The results of this study provide evidence that among the parental involvement variables examined, only mother-child communication was positively related to middle school students’ academic performance in the subjects of Chinese, mathematics, and English. The findings suggest that compared to parental non-academic activity involvement, parent-child communication has a more pronounced impact on the academic performance of middle school students (the main-effect model). Within parent-child communication, mother-child communication has a stronger effect on students’ academic performance than father-child communication. The findings of this study are in line with the research by Hsu et al. [13], who emphasize the importance of mother involvement in their study. Moreover, these results align with the understanding that mothers and fathers may play different roles within the family. For example, mothers are often associated with caretaking, while fathers are associated with play [44]. Notably, in Chinese society, fathers are generally regarded as financially responsible for the family and have less involvement in their children’s schooling compared to mothers [13, 45]. This cultural difference could contribute to the observed difference in the effect of mother-child communication and father-child communication on the academic performance of Chinese middle school students. However, it should be noted that due to data availability, this study was unable to investigate the effect of mother non-academic activity involvement, father non-academic activity involvement, and other types of parental involvement. Future studies that examine different types of parental involvement and differences between mothers and fathers are recommended to advance our understanding in this field.

The moderation effects of parental involvement were observed to have an impact on the relationship between middle school students’ mental unwellness and their academic performance in both Chinese and English. Specifically, when students perceived a higher level of mother-child communication, the negative relationship between their mental unwellness and their academic performance in Chinese was weaker. In the meantime, when students perceived a higher level of father-child communication, the negative relationship between their mental unwellness and academic performance in English was weaker. Because parental involvement and parental support share similarities [19–21], the aforementioned findings support the buffering model, which posits that social support can protect individuals from the negative effects of life stress [17, 18, 46, 47]. Accordingly, higher levels of parent-child communication can act as a protective factor for students who experience mental unwellness, shielding them from academic failure. However, the reason why mother-child communication appeared to be more effective in alleviating the inverse relationship between mental unwellness and academic performance in Chinese, while father-child communication appeared to be more effective in alleviating the negative relationship between mental unwellness and academic performance in English, remains unclear. Therefore, it is suggested that future studies, particularly qualitative in nature, be conducted to explore the underlying mechanisms at play.

### The effects of other variables

The findings of this study indicate that better prior academic performance, being female, and parental postsecondary education attainment were related to middle school students’ better academic performance in the subjects of Chinese, mathematics, and English. In the meantime, school location was found to have a differential impact on students’ academic performance across subjects. Results indicate that, compared to students from schools located in central urban/town areas, those from rural-urban fringe zones and rural areas exhibited significantly lower academic performance in mathematics and English. Additionally, students from schools located in towns outside of central urban/town areas displayed a lower level of English performance compared to their peers in central urban/town schools. These disparities could be attributed to socio-economic differences between developed and less developed areas. In China, students from urban areas generally enjoy better education quality than students from other areas [48]. Therefore, it is not surprising that middle school students from urban areas tend to have better academic performance than their counterparts from other areas. However, further research is needed to confirm these findings and explore the underlying reasons for these disparities.

The results of this study indicate a lack of correlation between school type and students’ academic performance in the subjects of Chinese and mathematics. On the other hand, school type was found to be associated with students’ performance in English, with public school students generally outperforming their private school counterparts. Nonetheless, private schools in China do vary. Because of the limited school sample size provided by the CEPS, this study grouped all types of private schools together, which masks the variability of the private schools. Therefore, the findings of the relationships between school type and students’ academic performance in Chinese, mathematics, and English should be interpreted with caution.

### Limitations

This study is subject to several limitations. First, this study has been limited to an examination of two types of parental involvement (namely, parental non-academic activity involvement and parent-child communication) due to the limitations in the available data, thus rendering the effects of other types of parental involvement untested. Second, the available data have also precluded an assessment of the differential effects of parental non-academic activity involvement by mothers and fathers, thereby failing to account for the unique impact of each parent. Third, this study does not establish causal relationships between students’ mental unwellness and their academic performance in Chinese, mathematics, and English. That is, while mental unwellness may predict students’ academic performance (adjustment erosion), academic performance may also predict mental unwellness (academic incompetence). Fourth, the CEPS measured students’ mental unwellness using its own scale rather than the other well-established scales such as the Generalized Anxiety Disorder 7-item scale (GAD-7) and the Patient Health Questionnaire depression scale (PHQ-9). Fifth, limited items in the CEPS can be used to measure parental non-academic activity involvement and parent-child communication. For example, parental non-academic activity involvement can also be measured by parents supporting their children to participate in extracurricular activities, while parent-child communication can also be measured by children talking to their parents about their future plans. Finally, the data utilized in this study are relatively dated, which may impair the generalizability of the results obtained.
